# Intralesional Vessel Diameter Measured by Optical Coherence Tomography Angiography Could Improve the Differential Diagnosis of Small Melanocytic Choroidal Lesions

**DOI:** 10.3390/cancers16122167

**Published:** 2024-06-07

**Authors:** Laura Vigués-Jorba, Daniel Lorenzo, Cristina Pujadas, Rahul Morwani, Liria Yamamoto-Rodriguez, Maria Baradad-Jurjo, Lluis Arias, Estefania Cobos, Pere Garcia-Bru, Juan-Francisco Santamaria, Olga Garcia Garcia, Josep-Maria Caminal

**Affiliations:** 1 Ophthalmology Department, Ocular Oncology Service, Bellvitge University Hospital, University of Barcelona, 08907 Barcelona, Spain; 2 Ophthalmology Department, Complex Hospitalari Moisès Broggi-Consorci Sanitari Integral, 08970 Barcelona, Spain; 3 Ophthalmology Department, University Hospital Joan XXIII, 43005 Tarragona, Spain

**Keywords:** choroidal melanoma, choroidal nevus, optical coherence tomography angiography, OCTA

## Abstract

**Simple Summary:**

The differential diagnosis between small melanocytic lesions and choroidal nevus is challenging. We usually rely on clinical risk factors to establish the need for immediate treatment vs. close monitoring followed by treatment if the lesion grows. We aimed to describe the features of small indeterminate choroidal melanocytic lesions visualized on optical coherence tomography angiography (OCTA) and to identify the predictors of growth. Our findings show that the vessel diameter quantified by OCTA can help differentiate between choroidal nevi and small melanomas, when considered together with clinical risk factors.

**Abstract:**

In this study, we aimed to identify the features of indeterminate choroidal melanocytic lesions visualized on optical coherence tomography angiography (OCTA) and to identify the predictors of growth. We retrospectively evaluated 86 patients with indeterminate lesions treated at our centre from 2016 to 2021. Clinical management involved active surveillance followed by brachytherapy if growth was detected. The lesions were classified into two groups according to whether they grew (small melanomas) or remained stable (choroidal nevi). Growth was detected in 19 (22.1%) lesions. All patients underwent OCTA at baseline. These images were compared to identify the possible predictors of growth. Significant between-group differences were observed in thickness (*p* = 0.00), greatest basal diameter (*p* = 0.00), number of risk factors (*p* = 0.00), symptoms (*p* = 0.001; relative risk [RR]: 4.3), orange pigment (*p* = 0.00; RR: 6.02), and ultrasonographic hollowness (Kappa sign); *p* = 0.000; RR: 5.3). The melanomas had significantly more vessels with a diameter ≥ 76.3 µm (*p* = 0.02; RR: 2.46). The time to growth in these lesions was significantly shorter (*p* = 0.05) than in lesions with smaller vessels. These findings show that vessel diameter quantified by OCTA can help differentiate between choroidal nevi and small melanomas, when considered together with clinical risk factors.

## 1. Introduction

Small choroidal melanomas and choroidal nevi share many features, and the differential diagnosis can be challenging. Nevertheless, it is essential to correctly identify choroidal nevi to avoid the unnecessary treatment of benign lesions, especially given the risk of treatment-related visual morbidity. Similarly, failure to diagnose a small melanoma promptly and accurately could have serious consequences, including lesion growth, increased metastatic potential, and a higher mortality risk [1]. Given the difficulty of accurately distinguishing between benign and malignant small melanocytic lesions, many authors support active surveillance [2,3,4,5,6]. This approach, which involves close monitoring of the lesion followed by treatment if it begins to grow, is associated with low mortality rates [7]. By contrast, other authors argue that lesions with clinical risk factors suggestive of malignancy should be treated immediately to prevent tumour growth and reduce the risk of metastasis [1,8,9,10].

Shields et al. [9] described several features—tumour characteristics, lesion size, presence of symptoms, and ultrasonographic imaging suggestive of malignancy—that are commonly used to determine the malignant potential of small melanocytic lesions. However, new prognostic factors are needed to facilitate the early identification of malignant lesions to improve clinical outcomes.

According to Folberg et al., the histopathological vessel pattern in choroidal melanomas has prognostic implications [11]. Those authors published a study in 1993 in which they predicted the emergence of new, noninvasive imaging techniques capable of detecting these vascular patterns, which they argued would provide valuable prognostic data. Clearly, their prediction was correct, as new imaging techniques such as optical coherence tomography angiography (OCTA) enable the visualization of tumour vascularization. OCTA can differentiate between static tissue and moving red blood cells, without the need for contrast agents [12]. In this regard, OCTA could be used to identify microvasculature patterns within melanocytic lesions, which could help to differentiate between benign and malignant lesions.

Although several previous studies have used OCTA to identify vascular patterns in choroidal melanocytic lesions [13,14,15,16,17,18,19], the use of this imaging technique remains relatively limited. Moreover, only a few studies have used OCTA to compare choroidal nevi and choroidal melanomas [20,21,22,23]. In this context, the aim of the present study was to describe and quantify the features of small choroidal melanocytic lesions visualized on OCTA and to identify the vascular factors that may help to identify malignant lesions or those with a high risk of progression.

## 2. Materials and Methods

This was an observational cohort study conducted at a tertiary referral centre in ocular oncology (Bellvitge University Hospital; Barcelona, Spain). This study adhered to the tenets of the Declaration of Helsinki, and the Clinical Research Ethics Committee at Bellvitge University Hospital approved the study protocol. All patient data were anonymized for this analysis.

We retrieved diagnostic OCTA images from 104 consecutive patients with small melanocytic choroidal lesions evaluated from May 2016 to March 2021. All lesions were stage T1a (8th edition, American Joint Committee on Cancer tumour staging criteria) [24]. Patients with a follow-up < 24 months or low image quality (motion or blinking artifacts) were excluded. A total of 86 patients met all inclusion criteria and were included in the study.

In all cases, the post-diagnosis treatment protocol was the same: active surveillance followed by treatment if lesion growth was detected during follow-up. Lesions that showed evidence of growth (defined as an increase of more than 0.3 mm in height or 0.5 mm in diameter in less than one year) were classified as small melanomas, while those that remained stable were classified as choroidal nevi. In accordance with routine clinical practice, the small melanomas were treated with brachytherapy.

A wide range of demographic and clinical variables were recorded, including the risk factors for lesion growth described by Shields et al. [25] (tumour thickness; subretinal fluid; orange pigment; symptoms; distance to optic nerve; absence of drusen or halo; and ultrasonographic hollowness), time to growth, and date of last follow-up.

**Figure 1:**
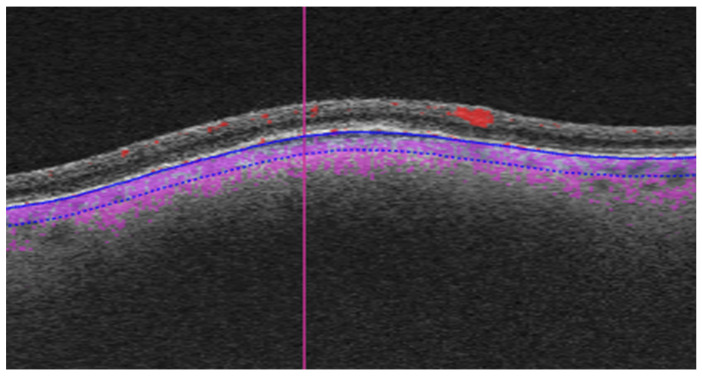
Manual segmentation of OCTA images. The choriocapillaris slab was expanded to a depth of 100 µm posteriorly to include the lesion (the analysed region is delimited by blue lines, purple dotting shows blood flow).

As part of the diagnostic protocol, all patients underwent OCTA scanning with Triton swept-source OCT (Topcon, Tokyo, Japan). We compared the OCTA images in the two groups (small melanoma vs. choroidal nevi) to identify any features that could help to predict the risk of growth. Most of the OCTA images were 6 × 6 mm, although some were 4.5 × 4.5 mm or 3 × 3 mm in accordance with the size of the lesion. We corrected for these differences by scaling the images during processing. The OCTA images were manually segmented by selecting the choriocapillaris slab, expanding it 100 µm, and moving it posteriorly to include the lesion (Figure 1) [26].

**Figure 2:**
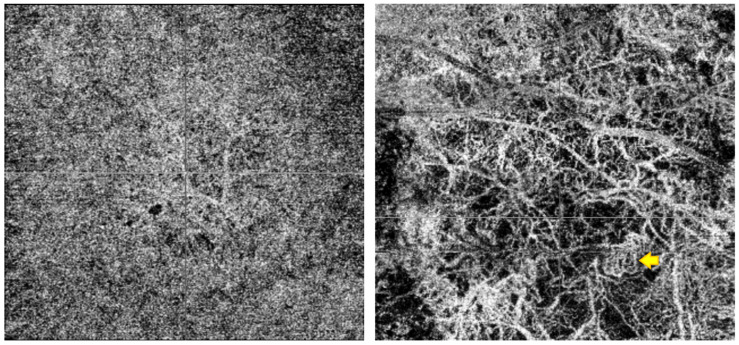
The (**left**) image is the AOCT of a lesion that remained stable during follow-up, with a fine, regular vascularisation pattern. To the (**right**), we can observe a lesion that grew, with larger vessels in the AOCT, avascular areas, and a complex vascularisation pattern with cross-links and loops (example shown by the yellow arrow).

**Figure 3:**
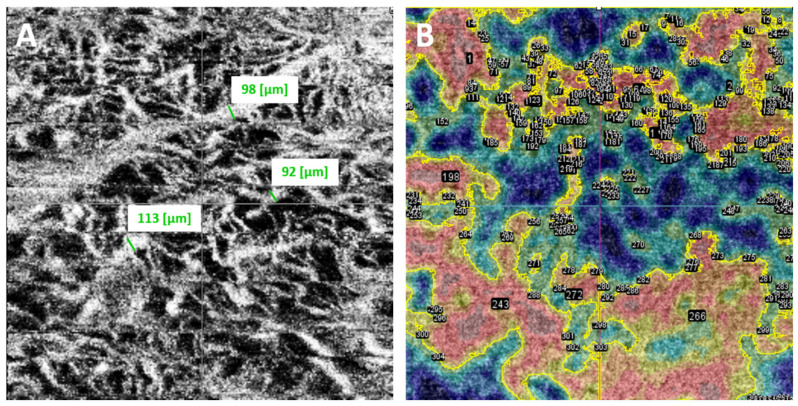
(**A**). Each independent observer measured the diameter of three different vessels, selecting the largest vessels distinguished on the en face images (obtained measurements are shown in green next to the selected vessels). (**B**). Using ImageJ software, vessel density maps were analysed. The areas of greatest vascular density were selected according to colour (shown surrounded by a yellow contour line) to calculate the percentage area occupied by high versus low vascular density.

Three independent observers (an ocular oncologist, a retina consultant, and a senior resident)—all blinded to the course of the lesions (growth vs. no growth)—evaluated the B-scan and en face OCTA images. The observers recorded the following OCTA-related variables, vascular flow inside the lesion and choriocapillaris (B-scan), as well as presence or absence of the following: hyperreflective ring; well-defined margin; vascularization and loops in the en face image (Figure 2). In case of disagreement, the observers reviewed the images again to reach consensus. Each observer manually measured the diameter of three different vessels on the en face images, selecting the largest vessels distinguished and avoiding, if necessary, shadowing vessels from the retinal vasculature (Figure 3A). The AngioTool v. 64 0.6a software [27] was used to process the en face OCTA images to obtain the percentage of the image covered by vessels (vessel percentage area), the junction density (number of vessel junctions per mm^2^), and lacunarity (a measure of the gaps between vessels). We used the FIJI platform [28] to transform the en face images to binary code in order to calculate the fractal box count. Fractal analysis can be used to assess the degree of complexity of biological structures. It has been used to analyse retinal vasculature in fundus photographs, fluorescein angiography images [29], and OCTA images [30]. We used the method described by Zahid et al. [30] for fractal analysis. Finally, we used the Image J software (version 1.54f) [28] to analyse density maps in order to select the areas with greatest vascular density and to calculate the percentage area of high versus low vascular density (Figure 3B).

### Statistical Analysis

Descriptive statistics were used to summarize the OCTA parameters. The patients were grouped according to lesion behaviour (quiescent vs. growth) during follow-up.

Pearson’s chi-square test was used to compare dichotomous variables. If the expected frequency was <5, Fisher’s exact test was used. For quantitative variables, the Shapiro–Wilk normality test, Skewness, and Kurtosis tests were used to determine normality distribution. Student’s *t* test was used to compare normally distributed quantitative variables. The non-parametric Mann–Whitney U test was used for variables with a non-normal distribution.

Kaplan–Meier curves were drawn to evaluate time to tumour growth. Receiver operating characteristic (ROC) curves were drawn for the largest vessel diameter. The intersection point between sensitivity and specificity was used to determine the optimal cut-off point for diagnostic suspicion of melanoma. Lin’s concordance coefficient was used to assess inter-observer agreement.

The variables significantly associated with melanoma on the univariate analysis were included in a multivariate (Cox regression) model.

The STATA program (Stata/BE 17.0) was used to perform the statistical analysis. Statistical significance was set at *p* < 0.05.

## 3. Results

Table 1 shows the characteristics of the 86 patients (49 women and 37 men). At diagnosis, the mean (standard deviation [SD]) age was 61.0 (13.9) years. The mean (SD) tumour thickness was 1.22 (0.72) mm, with a maximum basal diameter of 5.91 (2.77) mm. Due to the OCTA image quality requirements, most lesions were located close to the fovea or optic nerve. The median (interquartile range [IQR]) distance to the optic nerve and fovea was 2.75 (4.2) mm and 1.20 (3.7) mm, respectively. The median (IQR) number of risk factors for progression at diagnosis was four (3). The most common risk factor (48% of cases) was a margin < 3 mm from the optic nerve. During follow-up, lesion growth was detected in 19 (22.1%) lesions. These were all treated with brachytherapy. Enucleation was performed in one case due to lesion recurrence. No cases of metastatic disease were observed. Three patients died during follow-up, all due to unrelated causes.

There were no statistically significant differences in age, sex, or initial visual acuity between the two groups at diagnosis. However, significant between-group differences were observed in several clinical variables. The melanoma group had significantly thicker lesions (*p* = 0.00), larger basal diameter (*p* = 0.00), more risk factors (*p* = 0.00), and more symptoms (*p* = 0.001; relative risk [RR]: 4.3, 95% confidence interval [CI]: 1.6–11.9). Significant between-group differences were also observed in the presence of orange pigment (*p* = 0.00; RR: 6.02; 95% CI: 2.2–16.6) and ultrasonographic hollowness (Kappa sign) (*p* = 0.000; RR: 5.3; 95% CI: 2.6–10.6) (Table 1). There were no between-group differences in the distance to the optic nerve or fovea, nor in the presence of subretinal fluid, drusen, or halo.

On the baseline OCTA scan, lesions in the melanoma group were significantly more likely to have a vessel diameter ≥ 100 µm (*p* = 0.012; RR: 3; 95% CI 1.4–4.3). There were no differences between the groups in the presence or absence of well-defined margins, a hyperreflective halo, vascular flow, or vascularization patterns (fine vessels or loops) (Table 2). No significant differences were observed in fractal box count, the proportion of high-flux areas, vessel percentage area, junction density, or mean lacunarity (Table 3).

On the ROC curve analysis, the optimal cut-off point to differentiate between melanomas and nevi on the baseline OCTA scan was 76.3 µm. Lesions identified as melanomas had significantly more vessels with a diameter ≥ 76.3 µm (*p* = 0.02; RR: 2.46) compared to the non-melanoma lesions. The sensitivity and specificity for this cut-off point were 47.4% and 80.6%, respectively, with a positive predictive value (PPV) of 40.9% and negative predictive value (NPV) of 84.4%. The melanomas contained significantly more vessels ≥100 µm (*p* = 0.01; RR: 3; 95% CI 1.4–6.3) compared to the choroidal nevi. Using this cut point, the sensitivity fell to 15.8% (versus 47.4% for the 76.3 µm cut-off) and specificity increased to 94.0% (vs. 80.6%). The PPV and NPV were 43% and 80%, respectively.

**Figure 4:**
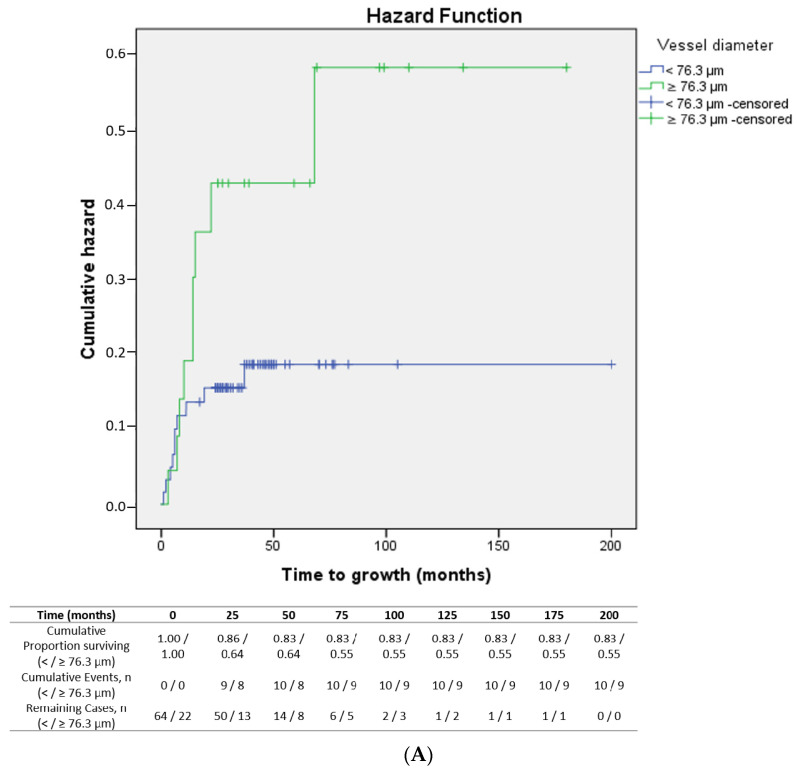

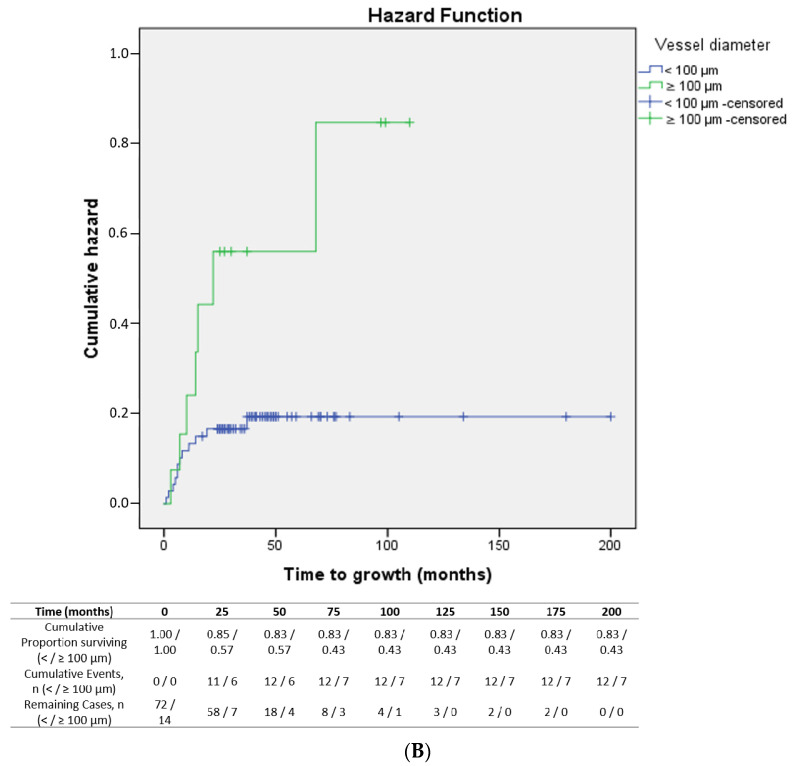
(**A**): Kaplan–Meier curve showing time to growth of lesions according to vessel diameter (< vs. ≥76.3 µm) (*p* = 0.05). (**B**): Kaplan–Meier curve showing time to growth of lesions according to vessel diameter (< vs. ≥100 µm) (*p* = 0.01).

The mean time to lesion growth in the melanomas was 8 months. The median total follow-up was 41.5 months. Lesions whose vessel diameter was ≥76.3 µm (Figure 4A) and ≥100 µm (Figure 4B) presented a significantly shorter time to growth (*p* = 0.05 and *p* = 0.01, respectively) compared to lesions with smaller vessel diameters.

**Figure 5:**
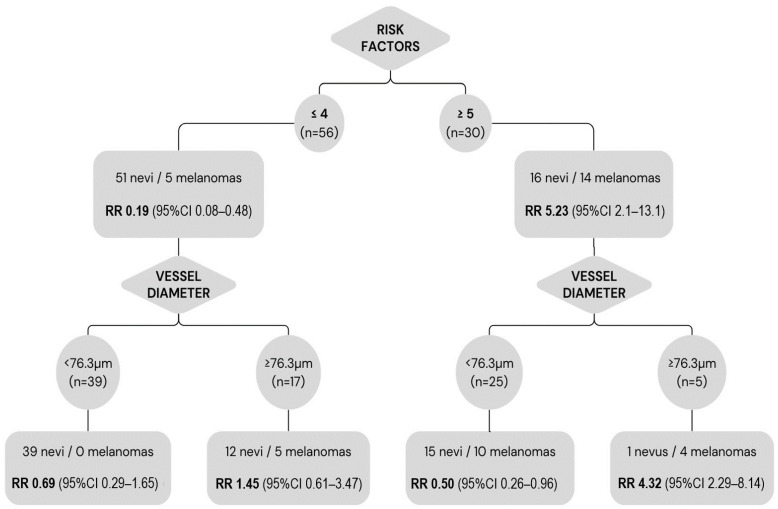
Relative risk of melanoma according to established clinical risk factors and greatest vessel diameter. Lesions with ≤4 risk factors at baseline had a significantly lower risk of melanoma (RR: 0.19); however, within this group, the presence of vessel diameters ≥ 76.3 µm implies a higher risk (RR: 1.45). Lesions with ≥5 risk factors had a higher risk of malignancy (RR: 5.23); nevertheless, the presence of vessels with diameters < 76.3 µm within this group was protective (RR: 0.5).

None of the lesions with <2 risk factors at diagnosis showed growth during follow-up. Among the lesions with ≤4 risk factors, the presence of vessels with diameters ≥ 76.3 µm was associated with a 29.4% higher risk of melanoma versus lesions with smaller vessel diameters (risk difference 0.294; 95%CI: 0.08–0.51). The relative risk of melanoma in lesions with ≥5 risk factors was 5.23 (95% CI: 2.08–13.11). In those lesions, the presence of vessels with diameters < 76.3 µm was protective (RR: 0.5; 95% CI: 0.26–0.95) (Figure 5).

The following variables were significantly associated with melanoma in the univariate analysis: lesion thickness, symptoms, orange pigment, ultrasonographic hollowness, and vessel diameter ≥ 76.3 µm. These variables were included in a Cox regression model. In that model, orange pigment (*p* = 0.005) and ultrasonographic hollowness (*p* = 0.003) remained significant predictors of malignancy.

The inter-observer agreement (Lin’s concordance correlation coefficient) for the measurement of the largest vessel diameter was 0.9.

## 4. Discussion

Numerous studies have evaluated the histopathological vessel structure in choroidal melanomas, and several different patterns have been identified. According to Folberg et al. [11], the prognostic factor most closely associated with poor outcomes is the presence of vascular networks. In this study, we used OCTA to analyse differences in vascular structure in small, indeterminate melanocytic lesions in an attempt to identify the prognostic factors that would allow for a high-probability diagnosis of melanoma, potentially offering an alternative to the “active surveillance” approach.

The main finding of this study is that the intratumoural vessel diameter measured by OCTA can help differentiate choroidal nevi from small melanomas, especially when the vessel diameter data are considered together with established clinical risk factors. Moreover, the integrated OCT software makes it easy to measure the vessel diameter, as evidenced by the good inter-observer concordance observed in our study (despite the disparity in experience level among the independent observers). Our findings suggest that the presence of large diameter vessels (≥76.3 µm) on OCTA indicates a higher risk of malignancy. This information is particularly useful in lesions with fewer risk factors (≤4), in which the risk of malignancy is normally considered to be lower.

Previous studies that have used OCTA to differentiate between nevi and melanomas have identified several features that could help to differentiate between these two clinical entities. Cennamo et al. [16] evaluated OCTA images from 116 choroidal tumours, finding that flat choroidal nevi had a normal choriocapillaris layer, while melanomas had a dense, irregular vascular network. Toledo et al. [18] found that the presence of a hyporeflective plexus and hyperreflective ring correlated with higher risk lesions. Ghassemi et al. [17] found that choriocapillaris vascular flow was lower in melanomas than in choroidal nevi. Those authors hypothesized that this difference in flow was due to the compression of the retinal pigment epithelium-Bruch membrane during melanoma growth. Garcia-Arumi Fuste et al. [21] compared the features observed on OCTA in 18 choroidal nevi and 18 melanomas, finding that choroidal melanomas were more likely to present border irregularity, choriocapillaris hyporeflectivity, hyperreflective ring, avascular areas, and vascular anomalies such as vascular loops and thick networks. Greig et al. [23] found that the presence of avascular areas and choroidal vessel visualization was associated with higher risk lesions. Those authors found that melanomas could be differentiated from nevi based on choroidal vessel depth, which was significantly deeper in melanomas. They also identified two vascular structures present in melanomas, which they referred to as “nevus-like vasculature” and “complex vasculature”, with loops and cross-linking. In a study by Gönen et al. [22], most (71.9%) of the choroidal nevi had a normal choriocapillaris layer. Avascular areas were present in only 6.2% of the nevi versus 90% of the melanomas. Vascular loops were observed in 60% of the melanomas and choroidal neovascularization in 21.9% of the choroidal nevi.

In our patient cohort, we did not find any significant between-group differences in choriocapillaris or intralesional vascular flow. By contrast, Greig et al. [23] found a reduced choriocapillaris flow in elevated nevi and melanomas compared to flat nevi but no differences when the nevi and melanomas were size matched, a finding that suggests that flow reduction is attributable to compression rather than to malignant potential. We did not detect any differences between the nevi and melanomas with regard to the presence or absence of a fine vascularization pattern, which we had expected to find in the nevi, nor in vessel loops, which we had expected to observe in the melanomas.

We used the Angiotools [27] and Fiji [28] software for image processing and to calculate the fractal box count, the proportion of high-flux areas, the vessel percentage area, junction density, and mean lacunarity. We had hoped that these tools would allow us to standardize image processing and automate measurements, which would save time and reduce observer error. However, although these programs are useful image processors, they were time-consuming and laborious and do not appear suitable for routine clinical practice. Although we did not observe any statistically significant differences between the groups in the aforementioned parameters, we did find a trend towards a lower vessel percentage area in the melanomas versus the nevi (*p* = 0.052), which we hypothesize could be due to increased avascular areas in melanomas, a finding that would be consistent with previous studies [22].

There were significant differences between the melanoma and nevi groups in terms of vessel diameters. Lesions with a larger vessel diameter on the baseline OCTA scan had a greater risk of becoming small melanomas. This finding is highly relevant, especially considering how easy it is to obtain this measurement using the integrated software. Moreover, inter-observer concordance for vessel diameter was very good, despite the large differences in clinical experience among the three observers. The optimal cut-off point to differentiate nevi from small melanomas was a vessel diameter of 76.3 µm. Lesions with a vessel diameter above this cut point had a 3.4 greater risk of melanoma. We performed a second analysis using a 100 µm cut-off point, as this cut-point is easy to visualize in the OCT software and could promote greater applicability in routine clinical practice. The sensitivity fell to 15.8% (versus 47.4% for the 76.3 µm cut-off) and specificity increased to 94.0% (vs. 80.6%). In short, our analysis indicates that the presence of large vessels (≥76.3 µm) in indeterminate melanocytic lesions increases the suspicion of malignancy (specificity: 80.6%). However, given the low sensitivity (47.4%), the absence of large vessels cannot rule out malignancy.

The vessel diameter measured on OCTA, considered together with the risk factors described by Shields et al. [25,31], provides valuable prognostic information (Figure 5). Lesions with ≤4 risk factors at baseline had a significantly lower risk of melanoma (RR: 0.19). However, this protective factor was lost within this group, in the presence of vessels ≥ 76.3 µm (RR: 1.45). In the group of patients with ≤4 risk factors, those with a vessel diameter ≥ 76.3 µm had a 29.4% higher risk of melanoma versus those with smaller vessels. Based on these findings, the presence of large-diameter vessels in indeterminate melanocytic lesions indicates that these lesions should be closely monitored, even if the lesion is considered low risk according to conventional criteria.

Not surprisingly, we found that lesions with ≥5 risk factors had a higher risk of malignancy (RR: 5.23) than lesions with fewer risk factors. In this high-risk group, the absence of vessels with diameters above the cut-off point (76.3 µm) was a protective factor (RR: 0.5). Although the presence of a small vessel diameter alone had a low PPV (40.9%) and cannot be used to rule out malignancy, the absence of large-diameter vessels in high-risk lesions suggests a lower risk of melanoma.

### Limitations and Strengths

The main limitation of this study is the limited number of melanomas (*n* = 19) compared to the number of choroidal nevi (*n* = 67), which limits the statistical power. In addition, due to the technical aspects required to obtain high-quality OCTA images, we only evaluated small tumours in the posterior pole, which means we do not know if these findings also apply to peripheral lesions. Another limitation is the potential for selection bias related to the type of hospital. Our hospital is a tertiary referral centre, which means that most of our patients have high-risk, indeterminate lesions. It is probable that the prevalence of melanoma in this cohort may be higher than observed in non-referral hospitals. Similarly, it may not be possible to extrapolate the PPV and NPV values to different settings.

To our knowledge, this is the first study to identify the quantified vessel diameter size to differentiate choroidal nevi from small melanomas. We also calculated the relative risk of developing melanoma based on the combination of clinical risk factors and vessel diameter, showing that this combination yields useful additional information to facilitate the differential diagnosis.

## 5. Conclusions

This study shows that the intralesional vessel diameter quantified by OCTA imaging can help differentiate between choroidal nevi and small melanomas. The determination of this parameter, considered together with established clinical risk factors, could improve the differential diagnosis. Crucially, the integrated OCT software makes it easy to measure the vessel diameter, with excellent inter-observer agreement. The presence of vessels with large diameters evidenced on OCTA may be particularly useful in lesions with few clinical risk factors that would otherwise be considered to have a low risk for melanoma.

## Figures and Tables

**Table 1:** Demographic and clinical characteristics.

Variable	Total (*n* = 86)	Nevi (*n* = 67)	Melanomas (*n* = 19)	*p* Value †	RR for Growth (95% CI) †
**Demographics**					
Age at diagnosis, mean (SD)	61.01 (13.9)	60.6 (15.1)	62.4 (9.1)	0.633 *	
Sex				0.538 **	0.77 (0.3–1.8)
Female, *n* (%)	49 (57.0)	37 (55.2)	12 (63.2)		
Male, *n* (%)	37 (43.0)	30 (44.8)	7 (36.8)		
Laterality				0.663 **	
Right eye, *n* (%)	46 (53.5)	35 (52.2)	11 (57.9)		
Left eye, *n* (%)	40 (46.5)	32 (47.8)	8 (42.1)		
Visual acuity, median (IQR)	0.9 (0.5)	0.9 (0.4)	0.8 (0.5)	0.369 ***	
**Small Melanocytic Lesions**					
Thickness (mm), mean (SD)	1.22 (0.72)	1.04 (0.67)	1.88 (0.46)	0.000 ***	
Greatest basal diameter (mm), mean (SD)	5.91 (2.77)	5.23 (2.55)	8.34 (2.13)	0.000 *	
Distance to the optic nerve (mm), median (IQR)	2.75 (4.2)	2.70 (4.6)	3.00 (2.3)	0.766 ***	
Distance to fovea (mm), median (IQR)	1.20 (3.7)	1.70 (4.2)	0.00 (2.8)	0.213 ***	
Number of risk factors present at diagnosis, median (IQR)	4 (3)	3 (2)	6 (1.5)	0.000 *	
Thickness > 2 mm, *n* (%)	12 (14.0)	6 (8.96)	6 (31.6)	0.021 ****	**2.85 (1.3–6.0)**
Subretinal fluid in optical coherence tomography, *n* (%)	45 (52.3)	32 (47.8)	13 (68.4)	0.112 **	1.97 (0.8–4.7)
Symptoms, *n* (%)	40 (46.5)	25 (37.3)	15 (78.9)	0.001 **	**4.3 (1.6–11.9)**
Orange pigment, *n* (%)	33 (38.4)	18 (26.9)	15 (78.9)	0.000 **	**6.02 (2.2–16.6)**
Margin ≤ 3 mm from optic nerve, *n* (%)	48 (55.8)	37 (55.2)	11 (57.9)	0.836 **	1.09 (0.5–2.4)
Ultrasonographic hollowness (Kappa sign), *n* (%)	15 (17.4)	5 (7.46)	10 (52.6)	0.000 ****	**5.26 (3.6–10.7)**
Halo, *n* (%)	2 (2.3)	2 (2.99)	0 (0.0)	1.000 ****	0.97 (0.9–1.0)
Drusen, *n* (%)	42 (48.8)	36 (53.7)	6 (31.6)	0.088 **	0.48 (0.2–1.2)
**Changes during Follow-Up**					
Lesion growth, *n* (%)	19 (22.1)	0 (0.00)	19 (100)		
Local recurrence after Brachytherapy treatment, *n* (%)	1 (1.16)	0 (0.0)	1 (5.3)	0.221 ****	
Metastasis, *n* (%)	0 (0.00)	0 (0.0)	0 (0.0)		
Death, *n* (%)	3 (3.5)	2 (3.0)	1 (5.3)	0.532 ****	
Follow-up (months), median (IQR)	41.5 (26)	41 (29)	44 (20)	0.337 *	

Abbreviations: SD, standard deviation; IQR, interquartile range; RR, relative risk. † Values marked in bold are statistically significant. * *t*-test. ** χ^2^ test. *** Mann–Whitney U test. **** Fisher’s exact test.

**Table 2:** Lesion characteristics observed on OCTA imaging.

Variable	Total (*n* = 86)	Nevi (*n* = 67)	Melanomas (*n* = 19)	*p* Value †	RR for Growth (95% CI) †
Largest vessel diameter (µm), median (IQR)	56.1 (31.1)	55.1 (28.0)	62.8 (47.2)	0.135 ***	
Largest vessel diameter ≥ 100 µm, *n* (%)	14 (16.3)	7 (10.4)	7 (36.8)	0.012 ****	**3 (1.4–6.3)**
Largest vessel diameter ≥ 76.3 µm, *n* (%)	23 (26.7)	14 (20.9)	9 (47.4)	0.025 ****	**2.46 (1.2–5.3)**
Well-defined margins, *n* (%)	23 (26.74)	20 (29.9)	3 (15.8)	0.222 **	0.51 (0.2–1.6)
Hyper-reflective ring, *n* (%)	28 (32.56)	23 (34.3)	5 (26.3)	0.511 **	0.74 (0.3–1.8)
Choriocapillaris vascular flow, *n* (%)	83 (96.51)	66 (98.5)	17 (89.5)	0.121 ****	**0.31 (0.1–0.8)**
Intra-lesional vascular flow, *n* (%)	47 (54.65)	34 (50.7)	13 (68.4)	0.172 **	1.80 (0.8–4.3)
Intra-lesional vascularisation, *n* (%)	85 (98.84)	66 (98.5)	19 (100)	1.000 ****	1
Fine vascularisation pattern, *n* (%)	74 (86.05)	59 (88.1)	15 (78.9)	0.452 ****	0.61 (0.2–1.5)
Vessel loops, *n* (%)	50 (58.14)	39 (58.2)	11 (57.9)	0.980 **	0.99 (0.4–2.2)

Abbreviations: IQR, interquartile range; RR, relative risk. † Values marked in bold are statistically significant. ** χ^2^ test. *** Mann–Whitney U test. **** Fisher’s exact test.

**Table 3:** Lesion characteristics assessed with the ImageJ and Angiotools software programs.

Variable	Total (*n* = 86)	Nevi (*n* = 67)	Melanomas (*n* = 19)	*p* Value †
	Median (IQR)			
Fractal box count, *n*	1.87 (0.04)	1.88 (0.03)	1.84 (0.04)	0.515
Proportion of high flux areas, %	47.0 (7.2)	47.0 (7.2)	47.4 (6.4)	0.525
Vessel percentage area, %	51.7 (3.2)	51.7 (2.5)	49.9 (4.4)	0.052
Junction density, junctions/mm^2^	0.0018 (0.001)	0.0019 (0.001)	0.0018 (0.002)	0.336
Mean lacunarity (Λ)	0.037 (0.02)	0.037 (0.02)	0.038 (0.02)	0.122

Abbreviations: IQR, interquartile range; † Mann–Whitney test.

## Data Availability

The raw data supporting the conclusions of this article can be made available by the authors on request.
